# Consciousness, not only intentionality, yields self-harming behavior

**DOI:** 10.3389/fnhum.2015.00069

**Published:** 2015-02-12

**Authors:** Reinhard Tschiesner, Demis Basso

**Affiliations:** ^1^Faculty of Education, Free University of Bozen-BolzanoBolzano, Italy; ^2^Centre for Applied Cognitive NeurosciencesRome, Italy

**Keywords:** consciousness, intentionality, assessment, self-harm, suicides, sensation-seekers

**A commentary on the Research Topic**

**Consciousness: from assessment to rehabilitation**

by Olivetti, M., Lancioni, G. E., Huenefeldt, T., and Laureys, S.

As pointed out by Searle (1987), subjectivity and intentionality have always been defined as basic characteristics of consciousness. Both of them are also needed in order to determine whether a person is a (self-)murderer or not: a legal report of the consciousness state should verify whether at that particular moment a person was subjectively and intentionally able to decide on his/her own actions. Despite the relevance of this assessment, at the moment the scientific community is not providing a unified definition of consciousness (Velmans, 2009). This lack is particularly relevant for psychopathology, since consciousness problems have been connected to several clinical pathologies. In the past, very scarce attention has been paid to the clinical assessment of consciousness, and research was mainly devoted to disorders of consciousness. However, assessment of consciousness has been acknowledged as being extremely relevant for psychiatric pathologies too, as a way of preventing self-harming and suicidal behavior. Several pathologies could result in suicidal behavior, such as schizophrenia (Bob and Mashour, 2011), substance-related disorders (Bortelote et al., 2004), post-traumatic stress disorder (O'Brien and Nutt, 1998), eating disorders (Favaro and Santonastaso, 1995), and others. Literature has usually divided people linked to self-harm into two branches: those who committed suicidal actions with a specific intention (intentional suicides, ISP), and those who died as a consequence of a risky behavior, without a clear suicidal intention (risky behavior people, RBP).

The ISP sample is mainly composed by people who are/were suffering from either major or mixed depressive episodes (Schneider, 2003; Bortelote et al., 2004). These patients usually focus attention on single aspects of their perception, concerning inner experiences (Ringel, 1953) or circumstances (i.e., confrontation with life or traumatic events as well as developmental tasks: Tschiesner et al., 2012). Patients of this group perceive and elaborate only specific contents according to their symptoms, and this may be viewed also as a restriction of consciousness (Ringel, 1953; Pöldinger, 1968). The content of their phenomenal consciousness consists, on the one hand, mainly of emotional experiences in many cases rated more intense as normal, and, on the other hand, of a constriction in perceiving alternative ways of behavior or reaction in difficult situations. Therefore, a dysphoric mood characterizes consciousness and triggers intentions of the same connotation, forcing people to direct aggression toward themselves (Ringel, 1953). Rottenberg et al. (2006) have shown this congruency between mood and behavior in depressive patients, a fact that, in turn, would explain why it is so difficult to defer their attention from their negative emotional status, which could lead them to carry out suicidal actions. Self-harming behavior is therefore one of the aims of ISP, and consistent with, and caused by, their constricted consciousness.

The RBP category includes people that are for example looking for thrills doing extreme sports or putting themselves in dangerous situations, or doing their official jobs like policemen, paramedics, soldiers, or firefighters (Gomà-i-Freixanet, 1995; Tschiesner, 2012). The drive for situations characterized by novelty and intensity has been described in the Sensations Seeking sub-construct “thrill and adventure seeking” (Zuckerman, 2007). Sensation-seekers are described as showing a low level of arousal, and they try to increase it, in an unconscious way, by doing extreme sports, consuming drugs, attending religious rituals and so on. This way of controlling arousal through intense and novel stimuli (Arnett, 1995; Zuckerman, 2007) has been indicated as the main indirect cause for assuming risks and provoking accidents. Accordingly, Schneider and Rheinberg (2003) argued that the aim of risky behavior—by doing risky activities–is not the danger in itself, but the ability to keep control in dangerous situations. Keeping control in a desperate situation like climbing a wall without a rope is linked with pleasure (Balint, 1959). Rephrasing, we may say that RBP seek to manage themselves in extreme situations, in which normal people wouldn't be able to do so. This way, RBP are intentionally targeting on the performance but not the correlated risks, the latter being part of the whole situation, but excluded from conscious analysis. The access to consciousness is influenced not only by vigilance (Overgaard and Overgaard, 2010), but also by the content itself: in RBP, only part of the intention is represented within consciousness (the one related to the proximal activity, but not the one related to arousal and to danger). One question that clinical practice poses to basic research concerns the mechanism that selects certain aspects and inhibits others from being accessed by consciousness.

Whereas harm and death in ISP are contents within consciousness, RBP do not include risk as a possible cause of harm or death. The former report to have intentionally attempted to carry out a suicidal action, the latter would deny it. These cases seem to differ with regard to the presence/absence of intentionality. The mood makes the difference: while ISP's mood is characterized by dysphoria and leads to suicidal behavior, RBP's mood is guided by pleasure felt in extreme situations. However, in our opinion, self-harmful behavior in both samples is influenced by the limited content in consciousness and determined by people's affection and needs, not only by vigilance.

The importance of consciousness and intentionality assessment has obvious implications on several applications, such as law (Rigoni et al., 2010). In our opinion, basic research on relevant clinical or subclinical samples like these could be very helpful for elucidating mechanisms of consciousness. In addition, this research would have an immediate impact on assessment and prevention of these self-harming behaviors.

## Conflict of interest statement

The authors declare that the research was conducted in the absence of any commercial or financial relationships that could be construed as a potential conflict of interest.

## References

[B1] Arnett J. (1995). The young and the reckless: adolescent reckless behavior. Curr. Dir. Psychol. Sci. 4, 67–71 10.1111/1467-8721.ep10772304

[B2] Balint M. (1959). Thrills and Regressions. London: Maresfield.

[B3] Bob P. Mashour G. A. (2011). Schizophrenia, dissociation, and consciousness. Conscious. Cogn. 20, 1042–1049. 10.1016/j.concog.2011.04.013 21602061

[B4] Bortelote J. M. Fleischmann A. DeLeo D. Wassermann D. (2004). Psychiatric diagnoses and suicide: revisiting the evidence. Crisis 25, 147–155. 10.1027/0227-5910.25.4.147 15580849

[B5] Favaro A. Santonastaso P. (1995). Suicidality in eating disorders: clinical and psychological correlates. Acta Psychiatr. Scand. 95, 508–514. 10.1111/j.1600-0447.1997.tb10139.x 9242846

[B6] Gomà-i-Freixanet M. (1995). Prosocial and antisocial aspects of personality. Pers. Indiv. Diff. 19, 125–134 10.1016/0191-8869(95)00037-7

[B7] O'Brien M. Nutt D. (1998). Loss of consciousness and post-traumatic stress disorder: a clue to aetiology and treatment. Br. J. Psychiatry 173, 102–104. 10.1192/bjp.173.2.102 9850219

[B8] Overgaard M. Overgaard R. (2010). Neural correlates of contents and levels of consciousness. Front. Psychol. 1:164. 10.3389/fpsyg.2010.00164 21887148 PMC3157935

[B9] Pöldinger W. (1968). Die Abschätzung der Suizidalität. Bern: Huber. 7453875

[B10] Rigoni D. Pellegrini S. Mariotti V. Cozza A. Mechelli A. Ferrara S. D.. (2010). How neuroscience and behavioral genetics improve psychiatric assessment: report on a violent murder case. Front. Behav. Neurosci. 4:160. 10.3389/fnbeh.2010.00160 21031162 PMC2965016

[B11] Ringel E. (1953). Der Selbstmord. Wien: Maudrich.

[B12] Rottenberg J. Hildner J. Gotlib I. (2006). Idiographic autobiographical memories in major depressive disorder. Cogn. Emot. 20, 114–128 10.1080/02699930500286299

[B13] Schneider B. (2003). Risikofaktoren für Suizid. Regensburg: Roderer.

[B14] Schneider K. Rheinberg F. (2003). Erlebnissuche und Risikomotivation, in Differentielle Psychologie und Persönlichkeitsforschung, 2nd Edn., ed Amelang M. (Göttingen: Hogrefe), 407–440.

[B15] Searle J. (1987). Minds and brains without programs, in Mindwawes, eds Blackmore C. Greenfield S. (Oxford: Blackwell), 208–253.

[B16] Tschiesner R. (2012). Sensation seeking, traumatic stress and coping: an empirical investigation in rescue forces. Neuropsychiatrie 26, 28–34. 10.1007/s40211-012-0002-1 22718420

[B17] Tschiesner R. Schweigkofler H. Favaretto E. Prossliner J. Lun S. Schwitzer J. (2012). About epidemiology of suicidal behavior: an investigation in the health-service district of brixen. Neuropsychiatrie 26, 121–128. 10.1007/s40211-012-0024-8 23055306

[B18] Velmans M. (2009). Understanding Consciousness, 2nd Edn. London: Taylor & Francis.

[B19] Zuckerman M. (2007). Sensation Seeking and Risky Behaviour. New York, NY: Maple-Vail Press 10.1037/11555-000

